# Breast and other cancers in 1445 blood relatives of 75 Nordic patients with ataxia telangiectasia

**DOI:** 10.1038/sj.bjc.6602658

**Published:** 2005-06-07

**Authors:** J H Olsen, J M D Hahnemann, A-L Børresen-Dale, S Tretli, R Kleinerman, R Sankila, L Hammarström, T E Robsahm, H Kääriäinen, A Bregård, K Brøndum-Nielsen, J Yuen, M Tucker

**Affiliations:** 1 Institute of Cancer Epidemiology, Danish Cancer Society, Strandboulevarden 49, DK-2100 Copenhagen; 2 The John F Kennedy Institute – National Eye Clinic, Gl. Landevej 7, DK-2600 Glostrup, Denmark; 3 Departments of Genetics, Institute for Cancer Research and University of Oslo, Faculty Division, Norwegian Radium Hospital, Montebello, N-0310 Oslo, Norway; 4 Cancer Registry of Norway, Institute of Epidemiological Cancer Research, Montebello, N-0310 Oslo; 5 Division of Cancer Epidemiology and Genetics, National Cancer Institute, 6120 Executive Blvd suite 7044, Rockville, MD 20852, USA; 6 Finnish Cancer Registry, Liisankatu 21 B, 00170 Helsinki, Finland; 7 Division of Clinical Immunology, Karolinska Institute, Huddinge Hospital, S-141 57 Huddinge, Sweden; 8 Department of Medical Genetics, University of Turku, Turku, Finland; 9 Department of Clinical Genetics, Turku University Hospital, Kiinamyllynkatu 10, FIN-20520 Turku, Finland; 10 Swedish University of Agricultural Sciences, S-750 07 Uppsala, Sweden

**Keywords:** ATM heterozygosity, early-onset breast cancer, cancer predisposition, familial cancer

## Abstract

Epidemiological studies have consistently shown elevated rates of breast cancer among female blood relatives of patients with ataxia telangiectasia (AT), a rare autosomal recessive disease. A large proportion of the members of AT families are carriers of AT-causing gene mutations in ATM (Ataxia Telangiectasia Mutated), and it has been hypothesised that these otherwise healthy carriers are predisposed to breast cancer. This is an extended and enlarged follow-up study of cancer incidence in blood relatives of 75 patients with verified AT in 66 Nordic families. Blood relatives were identified through population registry linkages, and the occurrence of cancer was determined from cancer registry files in each country and compared with national incidence rates. The ATM mutation carrier probabilities of relatives were assigned from the combined information on location in family, consanguinity, if any, and supplementary carrier screening in some families. Among the 1445 blood relatives of AT patients, 225 cancers were observed, with 170.4 expected, yielding a standardised incidence ratio (SIR) of 1.3 (95% confidence interval (CI), 1.1–1.4). Invasive breast cancer occurred in 34 female relatives (SIR, 1.7; 95% CI, 1.2–2.4) and was diagnosed in 21 women before the age of 55 years (SIR, 2.9; 95% CI, 1.8–4.5), including seven mothers of probands (SIR, 8.1; 95% CI, 3.3–17). When the group of mothers was excluded, no clear relationship was observed between the allocated mutation carrier probability of each family member and the extent of breast cancer risk. We concluded that the increased risk for female breast cancer seen in 66 Nordic AT families appeared to be restricted to women under the age of 55 years and was due mainly to a very high risk in the group of mothers. The findings of breast cancer risk in mothers, but not other likely mutation carriers, in this and other studies raises questions about the hypothesis of a simple causal relationship with ATM heterozygosity.

While mutations of both alleles of the ATM (Ataxia Telangiectasia Mutated) gene cause the rare autosomal recessive disorder ataxia telangiectasia (AT), heterozygous carriers of an ATM allele are healthy. Several studies, however, have estimated carriers to be at three- to five-fold increased risk for developing breast cancer (Swift *et al*, 1991; Athma *et al*, 1996; Inskip *et al*, 1999; Janin *et al*, 1999; Geoffroy-Perez *et al*, 2001; Olsen *et al*, 2001) and perhaps other cancers. With calculated mutation carrier frequencies in the general population in the order of 0.5–1% for ATM gene mutations, ATM heterozygosity might be responsible for a sizeable proportion of breast cancers in the (female) population (Easton, 1994).

We previously published a cohort study of cancer incidence in 1218 blood relatives of 56 Nordic AT patients from 50 families (Olsen *et al*, 2001). In order to increase the statistical power of the study, we added 16 more families and 227 relatives. We extended the follow-up for cancer incidence for 5 more years in Finland and Sweden and 7 more years in Denmark and Norway. We further corrected the mutation carrier probabilities based on supplementary mutation carrier testing of relevant members in some of the families. The results of this updated study are reported here.

## MATERIALS AND METHODS

In each of the participating countries (Denmark, Finland; Norway and Sweden), paediatric neurologists, paediatric immunologists, medical geneticists, cytogenetic laboratories and institutions for disabled children were requested to report cases of verified or suspected AT (from 1950 through 2002) to the country's study coordinator. The medical records were reviewed with respect to absolute and supporting criteria for the clinical diagnosis AT, as previously described (Olsen *et al*, 2001). Blood samples, lymphoblastoid cell lines or fibroblasts were available for most families, either from the proband (or affected siblings) when alive or from the parents. The ATM gene was screened for disease-causing mutations by heteroduplex analysis using DHPLC or protein truncating testing with subsequent sequence analysis at the cDNA and genomic level to identify the nature of the mutation (Laake *et al*, 2000; Bernstein *et al*, 2003). When biological samples were not available, the diagnosis of AT was based on the clinical and laboratory criteria.

Tracing of relatives for construction of pedigrees was based on data from the computerised national civil registration systems of the Nordic countries, as these systems make use of the personal identification number (PIN), unique for each citizen, allowing accurate linkage of registry information on parents and their offspring. These registration systems were started in 1960 in Norway, 1961 in Sweden, 1967 in Finland and 1968 in Denmark, when the PIN was assigned to all citizens alive at that date; for individuals born after that date, the PIN is assigned at birth. Information on more distant ancestors was derived from manual local population and church registers. Finally, follow-up information on date of death or emigration of blood relatives was obtained from the aforementioned national civil registration systems and from the national mortality files. Additional details are given in the previous publication (Olsen *et al*, 2001).

Data on blood relatives of AT patients were linked to the national cancer registry of the respective Nordic country by the subjects' PIN or, if they had died before the civil registration systems were computerised, their date of birth, date of death and name (Olsen *et al*, 1993). The period of follow-up for the occurrence of cancer among siblings, cousins, uncles, aunts and grandparents' siblings extended from the date of birth or the inception of national cancer registration (Denmark, 1943; Norway, 1953; Finland, 1953; Sweden, 1958), whichever came later, to the date of death or emigration or the end of study (31 December 2000 in Finland and Sweden and 31 December 2002 in Denmark and Norway), whichever came first. Similar rules were applied to the parents, grandparents and great-grandparents of the AT patients, except that follow-up was started at the earliest from the date of birth of the individual who was in direct line to the proband (e.g., the date of birth of the parent of the proband for grandparents). The malignant neoplasms identified in the cohort of relatives were classified according to the International Classification of Diseases, 7th Revision. The registration and coding practices of the four cancer registries have been described elsewhere (Tulinius *et al*, 1992).

### Statistical analysis

The expected numbers of cancers were calculated by multiplying the number of person-years of family members by the national cancer incidence rates for men and women in 5-year age groups and calendar periods of observation. Observed and expected numbers of cancers were pooled among countries, and standardised incidence ratios (SIRs), taken as the ratio of observed-to-expected cancers, were determined. The 95% confidence intervals (CIs) of the SIRs were calculated assuming a Poisson distribution of the observed cancers (Bailar and Ederer, 1964).

Cancer risk analyses were also undertaken after stratifying the study population according to their estimated gene carrier probability (1.0, 0.67, 0.5, 0.25, background). The individually assigned and estimated probability was the product of the location of the particular relative in the family pedigree (taking into account any information on consanguinity in the family) and the outcomes of any gene testing performed on members of the family, in addition to that already conducted on the proband, affected siblings and/or parents. For instance, if a grandmother tested positive for the one of the mutations of the proband, this changed the likelihood that the grandmothers' and the grandfathers' ancestors had been mutation carriers. In order to avoid bias due to selective testing of survivors in the families, however, the mutation carrier probability of the tested relatives themselves (which often was conducted years after the entry of the relative into the study cohort) were kept unchanged in the risk analyses. The number of actual mutation carriers was estimated by multiplying the probability of being a carrier by the number of subjects in each subgroup of female relatives. On this basis, the relative risk for female breast cancer associated with heterozygosity for ATM mutation was roughly estimated assuming that the excess risk for breast cancer observed in the entire group of female relatives can be ascribed to the subgroup of mutation carriers only (Olsen *et al*, 2001).

## RESULTS

A total of 75 AT patients from 66 families (24 patients from 21 families in Denmark, six patients from six families in Finland, 21 patients from 19 families in Norway and 24 patients from 20 families in Sweden) were included in the study. In nine families, two siblings were affected. The patients were born in the period 1949–2002 and all had a diagnosis of AT on the basis of clinical and laboratory findings. Biological material was available from the proband, an affected sibling and/or the parent(s) in 54 of the families representing 62 AT patients. A disease-causing mutation of the ATM gene was identified in both alleles in 57 patients from 50 of these families, while in five patients from four families material was available from only one parent, and therefore only one mutation was identified. In the remaining 13 patients from 12 families, biological material was not available, and the diagnosis was based entirely on clinical and laboratory information from the medical records. In two patients (from different families) who had both their mutations identified, the mutation of the maternal allele was shown to be *de novo*. Consequently, in the risk analyses stratified by carrier status, these mothers and their ancestors were regarded as carriers of a wild-type AT allele. The pedigrees indicated consanguinity in eight (12%) of the 66 families (mainly cousin–cousin marriages); however, homozygosity in the proband for a specific mutation was seen in 19 (38%) of the 50 families for which full allelic information was available. Of these, seven were a specific Norwegian founder mutation, the so-called Rendal mutation, located in exon 24 (3245delATCinsTGAT) (Laake *et al*, 2000).

In addition to gene testing of affected siblings and parents of probands, testing was conducted for 37 relatives from 13 Norwegian families: 21 on the basis of a blood sample from a live relative and 16 on the basis of tissue from a paraffin-embedded tumour block from a deceased relative. This increased the number of obligate carriers (from 144 to 152) as well as the numbers of relatives with gene carrier probabilities of 0.5 (from 441 to 459) and ‘background’ (from 42 to 136). On the contrary, it reduced the numbers of relatives with gene carrier probabilities of 0.67 (from 75 to 73) and 0.25 (from 873 to 755).

Of the 1575 unaffected blood relatives successfully identified, 130 had died before the date of eligibility, leaving 1445 relatives for cancer risk analysis. These consisted of 733 men and 712 women; 128 parents, 84 siblings, 189 grandparents, 241 uncles and aunts, 400 cousins, 170 great-grandparents and 233 grandparents' siblings. The entire group represented some 46 000 person-years of follow-up (mean, 31.7 years; range, 0–60 years), during which time 225 cancers were observed (106 in men and 119 in women), with 170.41 expected, yielding statistically significant SIRs of 1.3 (95% CI, 1.1–1.4) overall, 1.2 (0.9–1.4) for men and 1.4 (1.2–1.7) for women.

Of the 119 cancers in women, 34 were of the breast (all unilateral), with 19.51 expected, yielding a statistically significant SIR of 1.7 (Table 1). The association with breast cancer was particularly strong in the group of 64 mothers (the mother was missing in each of two Swedish families), who *a priori* were assumed to be ATM mutation carriers, although genetic testing revealed that two were not (SIR of 6.7 and a lower limit of the 95% CI of 2.9). In the remaining, combined group of female relatives, the SIR for breast cancer was a modest 1.4 and nonsignificantly increased, although with an indication of an excess risk for grandmothers, grandmothers' sisters and great-grandmothers. Table 1 also shows the risk for breast cancer according to estimated mutation carrier probability. Among the 10 women (mostly grandmothers) deemed to be mutation carriers by virtue of pedigree position or genetic testing, in addition to the 62 mothers being obligate carriers, there were no cases of breast cancers, decreasing the overall SIR of obligate carriers to 4.4, which still represents a significant risk elevation. The combined group of likely mutation carriers (probabilities of 0.67, 0.50 and 0.25) had a marginally significant, modest 60% increase in the risk for breast cancer, but no increase in risk with higher likelihood of being a mutation carrier (Table 1). On the basis of the carrier probability distribution given in the lower part of Table 1, 287 of the 712 female relatives included in the analysis were estimated to be ATM mutation carriers, that is, 40%. Assuming the existence of a true link between a mutated ATM allele and breast cancer, our data indicate that ATM heterozygosity on average infers a 2.9-fold (95% CI, 1.9–4.4) increase in the risk for female breast cancer.

Of the 34 cases of breast cancers among women, 21 were diagnosed before the age of 55 years (SIR, 2.9) and 13 at the age of 55 years or older (SIR, 1.1), suggesting the occurrence of early-onset breast cancer in these families (Table 2). Again, the association was clearly strongest for the mothers, with an 8.1-fold increase in risk in the age range below 55 years and a 3.3-fold increase as the lower 95% CI. Significantly increased risks were also seen for grandmothers, grandmothers' sisters and great-grandmothers when data were available for that age range. Nevertheless, detailed data on risk elevation by mutation carrier probability did not reflect an increasing risk with higher likelihood of being a carrier (Table 2).

No cases of breast cancer were observed among male relatives (0.1 expected).

When we analysed the risk for breast cancer by selected characteristics of the probands and the families, we did not observe any tendency to higher risks for breast cancer among female relatives, including mothers, in the eight reported consanguineous families (SIR, 1.4; 0.7–2.6), in the 19 families with probands homozygous for a mutation (1.3; 0.5–2.6), including the seven with Rendal mutation (1.5; 0.5–3.5), or in the nine families of probands with cancer (1.8; 0.7–3.7), than we did among female relatives of the remaining groups of families, yielding SIRs (95% CIs) of 1.8(1.2–2.7), 1.9 (1.3–2.8), 1.8 (1.2–2.6) and 1.7 (1.1–2.5), respectively. Among the nine mothers with two affected offspring, one case of breast cancer was observed (SIR, 4.3; 0.1–24), and among the remaining mothers with one affected child, seven cases were observed (SIR, 7.2; 2.9–15).

A total of 191 cancers were observed at sites other than the breast (106 in men and 85 in women) in the combined group of relatives, yielding an SIR of 1.2 (Table 3). The slightly but significantly increased overall risk was significant in women (SIR, 1.3) but not in men (1.2). As seen from the table, there was a tendency for increased risks for cancers at most sites, but malignant melanoma of the skin and cancer of the liver and biliary passages were the only sites for which the increase reached statistical significance. Six of eight liver and biliary passage cancers were observed only in female patients (SIR, 3.9; 95% CI, 1.4–8.4). The excess of malignant melanoma was seen primarily in the Norwegian subcohort with five observed cases, of which four were in males; interestingly, all five Norwegian cases were seen in families affected by the Rendal mutation (SIR, 5.4; 95% CI, 1.8–13). In the combined material, all six male cases (Table 3) were diagnosed in the age group of 60 years or above. There was no correlation to their likelihood of being mutation carriers; three of the 10 cases were seen in the subgroup with ‘background’ mutation carrier probability, when 0.6 was expected. Owing to the known genetic link between cancers of the breast and ovary, we reviewed family details for the eight AT family members with cancer of the ovary (SIR, 1.7). Two cases occurred in aunts, three in grandmothers and three in grandmothers' sisters, but no case occurred in mothers. There was no indication of an increased risk with higher likelihood of being a mutation carrier. Of eight cases of cancers of the liver and biliary passages, six were observed in women, yielding a significantly increased SIR of 3.9; of these, three were in the mutation carrier probability group 1 (0.08 expected; SIR, 36; 95% CI, 7.3–106), none in probability group 0.67/0.5 (0.5 expected), two in probability group 0.25 (0.8 expected) and one in probability group ‘background’ (0.2 expected). Two cases were seen in men (one in probability group 1 and one in group 0.50) when 0.8 was expected.

In contrast to the findings for breast cancer, the tendency to increased risks for cancers at other sites was not further strengthened when the analysis was restricted to persons aged below 55 years (SIR, 1.2; 95% CI, 0.9–1.7) compared to that of the entire study population (Table 3).

## DISCUSSION

In this extended and enlarged follow-up study of cancer incidence in 1445 blood relatives of 75 patients with AT, we observed a statistically significant, 2.9-fold increase in the risk for breast cancer among women under the age of 55 years and a risk close to that of the general female population for women in the age group 55 years or more. Our observation of an increased risk for early-onset breast cancer, now on the basis of 21 observed cases, corroborates the finding of our initial follow-up study, which was based on 13 cases (Olsen *et al*, 2001). The excess risk for breast cancer was evident in the mothers of the probands but less conspicuous in other female relatives, even those aged below 55 years. Although ATM heterozygosity in relatives on average was estimated to infer a significant 2.9-fold increased risks for breast cancer, if causal, our data did not convincingly point to a trend of increasing risk with each increment in the probability of being an ATM mutation carrier. These derived risk estimates on the potential role of ATM heterozygosity were to a large extent driven by the highly increased risk for breast cancer seen in mothers of probands. Although the absence of a clear correlation with the likelihood of being a carrier may be due to the small number of breast cancer cases in each probability group, it does appear to detract from the hypothesis of a causal link between the ATM mutation and breast cancer and raises questions about the likelihood of a simple genetic relationship.

The strengths of our study include the cohort design, the identification of study subjects from medical records, the unbiased identification of relatives through population registry linkage, the unbiased ascertainment and validation of cancer through cancer registry linkage, and the long (maximum, 60 years) and nearly complete follow-up of the entire study population. No family in the study was selected due to sporadic ATM gene mutation analyses of tumour tissue from cancer patients in the general population. The gene testing performed on tumour blocks from relatives affected with cancer was carried out after the identification of the patient and the relatives. Therefore, only the gene probability score of the ancestors of the tested person changed, and not the score of the person him- or herself. This avoided a bias due to selection of study subjects for testing that by definition had a cancer.

The observed excess risk for breast cancer among mothers is so large that neither chance nor confounding is a feasible explanation. Confounding would be possible if the mothers or other female relatives were less likely than the general population to have children or more likely to have children later in life (Ewertz *et al*, 1990). The reproductive pattern of the 66 families under study did not, however, indicate that either factor is of importance. On the contrary, we may have underestimated the strength of the association in female blood relatives in direct line with the AT patient (mothers, grandmothers and great-grandmothers), because the national rates of breast cancer are influenced by an approximately 30% higher risk for breast cancer among nulliparous women than among parous women. Confounding could also arise if the mothers of children with AT were more likely to undergo screening examinations for the early detection of breast cancer than the general population, because clinicians might be aware of the suggested link between ATM heterozygosity and breast cancer. This suggestion is, however, recent and is not yet widespread knowledge among colleagues or in affected families.

Large studies of blood relatives of patients with AT from France (Janin *et al*, 1999), the UK (Inskip *et al*, 1999) and the USA (Swift *et al*, 1991; Athma *et al*, 1996) have consistently shown an increased incidence or mortality (UK study) of breast cancer among female family members. In a cross-sectional analysis of 33 cases of breast cancer diagnosed in female relatives of 99 AT families in the USA, in which gene mutations were analysed, Athma *et al* (1996) found a significantly increased odds ratio of 3.8 for breast cancer among ATM gene carriers compared with noncarriers. This is compatible with our estimate of a 2.7-fold increased risk for breast cancer among female mutation carriers in general. The analysis of the US family data indicated, however, that the risk was increased among older women (⩾60 years) in particular, which clearly contrasts with our observation of an increased risk for early-onset breast cancer. Our finding does, however, appear to concur with that of the French study, which based on 29 observed female breast cancer cases in blood relatives of 34 AT families found the excess risk for breast cancer to be higher among female relatives below the age of 45 years than among female relatives above that age (Janin *et al*, 1999; Geoffroy-Perez *et al*, 2001). Also, as in our study, the risk for breast cancer among female relatives in the French study seemed to be restricted to the subgroup of presumed obligate carriers (including the mothers of probands), with five observed cancers among young carriers, equivalent to a significantly increased relative risk of 4.6. These findings are compatible with the estimate of 7.1 among young (<55 years) obligate carriers seen in our study. In the French study, the risks for breast cancer in the subgroups with estimated carrier probabilities of 0.67, 0.50 and 0.25 were similar to that of the general population, also indicating the absence of a clear relationship between mutation carrier probability and breast cancer risk. Unfortunately, the risk for breast cancer among mothers was not given separately. In the study in the UK, only mothers (obligate carriers) and grandmothers (0.50 probability carriers) were included as female relatives of the 95 AT probands (Inskip *et al*, 1999). On the basis of three observed deaths from breast cancer in each group of female relatives, the risk of mothers was nonsignificantly increased (SMR, 3.4) and that of grandmothers was close to that of the general population (SMR, 0.9), providing little support to a relationship between risk and carrier likelihood.

Thus, to our minds, the combined data from the published studies of breast cancer risk in female relatives of AT patients demonstrate a substantial and consistent increase in the risk for mothers. The existing international data on the risks for breast cancer of other female relatives are, however, still not conclusive, and convincing data to support a simple relationship between likelihood of ATM heterozygosity and risk of breast cancer has not yet been presented. Our extended data from the Nordic study showed no indication of variation in the risk for female breast cancer in analyses stratified according to the major characteristics of the probands or the families. An alternative hypothesis for the absence of a gradient of breast cancer incidence by increasing probability of being a gene carrier and the finding of an increased incidence mainly confined to mothers might be that giving birth to an AT child or having a pregnancy with a foetus affected with AT changes the mother's breast cancer risk – in combination with or regardless of any effect of her ATM heterozygosity. One can speculate whether microchimerism during pregnancy, that is, the phenomenon that foetal cells may pass into the maternal circulation and tissues, play a role in the highly increased risk of breast cancer seen among the mothers giving birth to an AT child. It has been suggested that microchimerism is associated with various immunological conditions of pregnancy and some chronic autoimmune conditions predominantly found in women (Bianchi, 2000), and in one study it has been associated with cervical cancer (Cha *et al*, 2003). It is conceivable that being pregnant with a foetus affected with AT may facilitate this biological phenomenon, and that the presence of foetal AT cells in the circulation or tissues of the mother may contribute to the development of maternal breast cancer. In our study, however, we saw no variation in breast cancer risk after stratification of mothers according to the number (one or two) of offspring affected by AT, although this conclusion is severely weakened by the small number of mothers and outcomes included. Detailed consistent data about pregnancies were not available in this study.

We found a slight increased risk for cancers at all sites except breast, which reached statistical significance for the female relatives only. There was a tendency for slight but nonsignificant elevations in risk for most diagnostic groups. Female relatives had excess numbers of cancers of the gall bladder and liver, which correlated with the mutation carrier probability of the subjects, but which was not replicated among male relatives. The slight increase seen for ovarian cancer was not correlated with mutation carrier probability or familial proximity to the proband. Similarly, there was no indication that the significantly increased risk for malignant melanoma in relatives of AT patients was correlated to their likelihood of being mutation carriers. The excess risk of malignant melanoma was due mainly to five observed cases (out of a total of 10) in the Norwegian subohort, all belonging to families affected by the Rendal mutation. Solar ultraviolet radiation is the main cause of malignant melanoma, and in Norway the incidence of this skin cancer varies markedly with latitude and elevation above sea level, for example, yielding a two-fold higher incidence of malignant melanoma in the south-eastern part of Norway compared to that of the northern part (Robsahm and Tretli, 2001). However, a review of the places of residence at diagnosis for the Norwegian relatives with malignant melanoma indicated that they were from areas in Norway with melanoma incidence rates close to the national average. Thus, we have no plausible explanation for this finding.

In one of their initial analyses of cancer incidence in blood relatives of AT patients, Swift *et al* (1991) reported a significant 2.5-fold increase in the risk for cancers at all sites combined in male relatives (73 observations) compared with that of spouses of female relatives (19 observations), and a significant 3.9-fold increase in all cancers in male obligate heterozygotes (18 observations). A risk estimate for all cancers other than breast in female relatives was not made in this study, but our recalculation on the basis of data reported in the paper indicates that the risk was much lower than that of male relatives. There is no good explanation for the observed difference between the two sexes in the US study, and there is no support in our study for a substantially increased risk for cancers among male relatives. In a separate analysis of the incidence of cancers at sites other than the breast, the French study obtained an overall RR of 0.9 for both sexes combined on the basis of 93 observations (Geoffroy-Perez *et al*, 2001). Liver was the only site for which there was a significant increase in risk, on the basis of six observed and 1.5 expected cases; sex-specific risks were, however, not given. Swift also reported two gall bladder cancers among obligate ATM mutation carriers, but he did not specify the sex of the cases nor present risk (Swift *et al*, 1991). Interestingly, there have been a few case reports of hepatocellular carcinoma in AT patients, all in female subjects (Kumar *et al*, 1979; Weinstein *et al*, 1985).

The main limitation of our study, as well as other studies of cancer in AT families, is small study groups with associated low precision in risk estimation for site-specific cancers, including female breast cancer, ovarian cancer, lymphomas and leukaemias. This indicates the need for increased international collaboration in the study of cancer in AT families and, if feasible, a combined analysis of the available study materials. The hypothesis of breast cancer risk related to being pregnant with an AT-affected child also needs to be pursued.

## Figures and Tables

**Table 1:** Standardised incidence ratios (SIRs) for breast cancer in 712 unaffected female blood relatives of 75 patients with ataxia telangiectasia (AT) from 66 Nordic families by familial relationship and probability of carrying an *ATM* mutation

			**Breast cancer**			
**Female relative**	**No.**	**Person-years at risk**	**Obs**	**Exp**	**SIR**	**95% CI**
All	712	22 892	34	19.51	1.7	1.2–2.4
						
Familial relationship						
Mother^a^	64	1330	8	1.20	6.7	2.9–13
Other female relatives	648	21 562	26	18.31	1.4	0.9–2.1
Sister of proband	39	871	0	0.05	—	—
Grandmother	94	3502	8	4.87	1.6	0.7–3.2
Aunt	121	5488	2	3.24	0.6	0.1–2.2
Great-grandmother	89	2340	8	3.89	2.1	0.9–4.1
Grandmother's sister	118	4357	7	5.43	1.3	0.5–2.8
Female cousin	187	5004	1	0.83	1.2	0.0–6.7
						
Mutation carrier probability^b^						
1^c^	72	1720	8	1.81	4.4	1.9–8.7
0.67; 0.50; 0.25	589	19 628	24	15.39	1.6	1.0–2.2
0.67; 0.50	252	9709	10	8.13	1.2	0.6–2.3
0.25	337	9919	14	7.33	1.9	1.0–3.2
Background^d^	51	1545	2	2.31	0.9	0.1–3.2

Obs=observed cancers; Exp=expected cancers; CI=confidence interval.

a The mother was not known in each of two Swedish families.

b The estimated mutation carrier probability on an individual, according to the location in the pedigree, including information on consanguinity combined with the outcome of any relevant gene testing of relatives (see also text).

c Two mothers regarded as carriers of a wild-type allele were excluded from this group.

d Relatives who married into consanguineous families, or relatives in branches of the family not involved in the gene transmission.

**Table 2:** Standardised incidence ratios (SIRs) for breast cancer in 712 unaffected female blood relatives of 75 patients with ataxia telangiectasia (AT) by age at diagnosis

	**Age at breast cancer diagnosis**							
	**<55 years**				**⩾55 years**			
**Female relative**	**Obs**	**Exp**	**SIR**	**95% CI**	**Obs**	**Exp**	**SIR**	**95% CI**
Total	21	7.19	2.9	1.8–4.5	13	12.32	1.1	0.6–1.8
								
Familial relation								
Mother	7	0.86	8.1	3.3–17	1	0.34	3.0	0.0–16
Grandmother and grandmother's sister	9	3.07	2.9	1.3–5.6	6	7.23	0.8	0.3–1.8
Great grandmother	3	0.57	5.3	1.1–15	5	3.32	1.5	0.5–3.5
Cousin and aunt	2	2.63	0.8	0.1–2.8	1	1.43	0.7	0.0–3.9
								
Mutation carrier probability^a^								
1	7	0.98	7.1	2.9–15	1	0.83	1.2	0.0–6.7
0.67; 0.50	5	3.55	1.4	0.5–3.3	5	4.51	1.1	0.4–2.6
0.25	8	2.14	3.7	1.6–7.4	6	5.18	1.2	0.4–2.5
Background	1	0.52	1.9	0.0–11	1	1.80	0.6	0.0–3.1

Obs=observed cancers; Exp=expected cancers; CI=confidence interval.

a 0.005–0.01, as in the background population.

**Table 3:** Standardised incidence ratios (SIRs) for cancer at sites other than the breast in unaffected relatives of patients with ataxia teleangectasia (AT), both sexes combined and each sex separately

	**Both sexes**				**Men**			**Women**		
**Site of cancer**	**Obs**	**Exp**	**SIR**	**95% CI**	**Obs**	**SIR**	**95% CI**	**Obs**	**SIR**	**95% CI**
All sites except breast	191	155.76	1.2	1.1–1.4	106	1.2	1.0–1.4	85	1.3	1.1–1.6
										
Stomach	17	12.07	1.4	0.8–2.3	11	1.5	0.7–2.6	6	1.3	0.5–2.9
Colon and rectum	25	21.31	1.2	0.8–1.7	14	1.3	0.7–2.1	11	1.1	0.5–1.9
Liver and biliary passages	8	2.97	2.7	1.2–5.3	2	1.4	0.2–5.1	6	3.9	1.4–8.4
Respiratory tract	24	19.38	1.2	0.8–1.8	20	1.3	0.8–2.1	4	0.9	0.2–2.3
Cervix uteri	6	4.51	1.3	0.5–2.9	—	—	—	6	1.3	0.5–2.9
Corpus uteri	5	4.25	1.2	0.4–2.8	—	—	—	5	1.2	0.4–2.8
Ovary	8	4.60	1.7	0.8–3.4	—	—	—	8	1.7	0.8–3.4
Prostate	17	16.06	1.1	0.6–1.7	17	1.1	0.6–1.7	—	—	—
Urinary tract	8	12.68	0.6	0.3–1.2	8	0.9	0.4–1.8	0	0.0	0.0–1.0
Melanoma of skin	10	4.81	2.1	1.0–3.8	6	2.7	1.0–5.8	4	1.6	0.4–4.0
Other skin	13	10.96	1.2	0.6–2.0	4	0.7	0.2–1.7	9	1.8	0.8–3.5
Lymphatic and haematopoietic tissues	13	12.33	1.1	0.6–1.8	7	1.0	0.4–2.1	6	1.1	0.4–2.5
Other and unspecified	37	29.83	1.2	0.9–1.7	17	1.0	0.6–1.7	20	1.3	0.8–2.1

Obs=observed cancers; Exp=expected cancers; CI=confidence interval.
